# SG-APSIC1105: The effectiveness of the infection control interventions in decreasing multidrug-resistant organism transmission in the Department of Neonatology of Hung Vuong Hospital

**DOI:** 10.1017/ash.2023.65

**Published:** 2023-03-16

**Authors:** Ngo Nhung, Tran Thi Thuy Hang, Ngo My Nhung, Ngo My Nhung, Ngo Thi Thanh Tham, Bui Thi Thuy Tien, Phan Thi Hang

**Affiliations:** Obstetrics and Gynaecology, Hung Vuong Hospital, Ho Chi Minh City, Vietnam; Infection Control Department, Hung Vuong Hospital, Ho Chi Minh City, Vietnam; Infection Control Department, Hung Vuong Hospital, Ho Chi Minh City, Vietnam; Infection Control Department, Hung Vuong Hospital, Ho Chi Minh City, Vietnam; Infection Control Department, Hung Vuong Hospital, HoChi Minh City, Vietnam; Neonatal Department, Hung Vuong Hospital, Ho Chi Minh City, Vietnam; Vice President, Hung Vuong Hospital, HoChi Minh City, Vietnam

## Abstract

**Objectives:** Infection control and prevention (IPC) is one of the most important factors in decreasing multidrug-resistant organism (MRDO) transmission. We evaluated the effectiveness of the IPC program in reducing the spread of MDROs at the Department of Neonatology of Hung Vuong Hospital. **Methods:** This research was conducted from April 2020 to September 2020 in the neonatology department in 3 phases: (1) We determined the compliance rate of hand hygiene and high-touch surfaces cleaning (via camera monitoring). (2) We conducted the following interventions: We developed specific cleaning protocols for the neonatology department. We provided training regarding MRDO transmission control and prevention for healthcare workers. We implemented a counseling program for active screening and isolation for hospitalized children; added isolation rooms for children with MRDO asymptomatic infections. And we improved feedback efficiency through an online group between the infection control and prevention team and the department of neonatology. (3) We re-evaluated compliance with hand hygiene practices and cleaning of high-touch surfaces, then we compared the rates of positive MRDO cultures before and after these interventions. **Results:** Before the interventions, 453 hand hygiene observations were recorded and 322 high-touch-surface cleaning observations were recorded. The hand hygiene compliance rate improved significantly from 33.2% to 85.5% (PR, 11.9; 95% CI, 7.4–19.3; *P*< .01). The high-touch-surface cleaning rate increased from 82.4% to 93.5% (PR, 3.1, 95% CI, 1.5–6.4; *P* < .01). The rate of high-touch surfaces being cleaned with proper technique increased from 38.5% to 87.9% (PR, 11.6; 95% CI, 6.3–21.3; *P* < .01). In total, 103 swab samples were positive for MRDOs by culture before and after the intervention. The rate of positive MRDO cultures decreased from 80.8% to 64.7% (*P* = .017). **Conclusions:** Enhancing hand hygiene and high-touch-surface cleaning compliance helped reduce MRDO transmission in the Department Neonatology of Hung Vuong Hospital.

